# Deciphering the Protective Interplay between Cancer and Alzheimer’s: Advancing Drug Repositioning Strategies for Alzheimer’s Disease Treatment

**DOI:** 10.1002/alz.087380

**Published:** 2025-01-09

**Authors:** Hong Zhao, Matthew Vasquez, Ju Young Ahn, Wenjuan Dong, Jianting Sheng, Zheng Yin, Stephen T.C. Wong

**Affiliations:** ^1^ Houston Methodist Cancer Center, Houston, TX USA; ^2^ The TT & WF Chao Center for BRAIN and Houston Methodist Neal Cancer Center, Houston Methodist Hospital, Houston, TX USA; ^3^ Houston Methodist Research Institute, Houston, TX USA; ^4^ Department of Radiology, Weill Cornell Medical College, New York, NY USA; ^5^ Brain and Mind Research Institute, Weill Cornell Medical College, New York, NY USA; ^6^ Weill Cornell Medical College, New York, NY USA

## Abstract

**Background:**

Global epidemiological studies involving over nine million participants have shown a 35% lower incidence of Alzheimer’s Disease (AD) in older cancer survivors compared to those without a history of cancer. This inverse relationship, consistent across recent studies with methodological controls, suggests that cancer itself, rather than cancer treatments, may offer protective factors against AD. This insight opens avenues for novel therapeutic strategies targeting early AD by harnessing cancer‐associated protective factors.

**Methods:**

To investigate the potential protective effect of cancer against Alzheimer’s Disease (AD), we developed “cancer‐in‐AD” mouse models. These models involved injecting a small number of breast cancer cells into young AD‐model mice (5xFAD) and monitoring amyloid plaque progression. Additionally, we introduced extracellular vesicles (EVs) from breast tumor‐bearing mice into similar AD models. Using spatial transcriptomics, we analyzed brain tissue gene expression and cell‐cell interactions, focusing on the astrocyte‐microglia‐oligodendrocyte network near amyloid plaques. This approach helped identify potential drugs for repurposing in AD treatment.

**Results:**

The study found a significant reduction in amyloid burden within the brains of the cancer‐in‐AD mouse models compared to age‐matched cancer‐free AD mice. The administration of EVs from cancer animal’s plasma to the AD mice prompted the release of various inflammatory cytokines and chemokines. A key discovery was an activated astrocyte‐microglia‐oligodendrocyte signaling network that regulates amyloid‐beta homeostasis in these mouse brains. Out of 49 FDA‐approved drugs identified to induce this cancer‐induced signaling, 11 showed promise in improving AD symptoms and reducing amyloid and tau accumulations, in both preclinical and clinical studies.

**Conclusions:**

The study reveals a notable decrease in amyloid levels in AD mice with cancer or exposed to tumor‐derived EVs, linked to immune system reprogramming and glial network activation. This supports the study’s drug repositioning approach and sets the stage for further research into the anti‐AD properties of these drugs, focusing on identifying crucial signaling elements for enhanced drug repositioning and combination treatment strategies.

